# Do markers of adiposity and glycaemia mediate the association between low carbohydrate diet and cardiovascular risk factors: findings from the UK National Diet and Nutrition Survey (NDNS) 2008–2016

**DOI:** 10.1136/bmjnph-2022-000551

**Published:** 2023-08-30

**Authors:** Cláudia Raulino Tramontt, Saad Mouti, Marjorie Lima Do Vale, Xunhan Li, Rajna Golubic, Sumantra Ray

**Affiliations:** 1 NNEdPro Global Institute for Food Nutrition and Health, Cambridge, UK; 2 Consortium for Data Analytics in Risk, University of California, Berkeley, California, USA; 3 Diabetes Trials Unit, Oxford Centre for Diabetes, Endocrinology and Metabolism, University of Oxford, Oxford, UK; 4 School of Biomedical Sciences, Ulster University at Coleraine, Coleraine, UK; 5 Fitzwilliam College, University of Cambridge, Cambridge, UK

**Keywords:** dietary patterns, biomarker

## Abstract

**Objectives:**

To examine the associations between low carbohydrate diet (LCD) and conventional cardiovascular risk factors and investigate whether these associations are mediated by body mass index (BMI), waist circumference (WC) and haemoglobin A1c (HbA1c) applying causal mediation analyses.

**Methods:**

We included 3640 adults aged 45–80 years from the UK National Diet and Nutrition Survey programme (2008–2016) with data on dietary intake, anthropometric and biochemical parameters. Four hypothetical interventions were examined: (1) LCD, (2) Low carbohydrate (LC) and high fibre diet (LCHF), (3) LC and high saturated fat diet (LCHS) and (4) LC and high unsaturated fat diet (LCHU). BMI and WC were used as markers of obesity. Biochemical markers included HbA1c, total cholesterol, high-density lipoprotein and low-density lipoprotein (LDL) cholesterol, triglycerides, systolic and diastolic blood pressure and C reactive protein (CRP). BMI, WC and HbA1c were used as a mediator of the effects. The analysis was adjusted for sociodemographic characteristic, smoking, estimated total energy intake, alcohol consumption and antihypertensive medication. To identify a potential causal effect of LCD on cardiovascular disease (CVD) risk, we estimated the average treatment effect, and corresponding p values and CI for the total, indirect and direct effect of the treatment on the outcome.

**Results:**

BMI, WC and HbA1c fully mediated the association between LCD and triglycerides and fully mediated the effects of LCHF on LDL, although BMI and WC were not sufficient to fully mediate the effects of LCHF on triglycerides and CRP. BMI alone fully mediated the effects of LCHS on HbA1c, triglycerides, LDL and CRP. None of these mediators explained the effect of LCHU on CVD risk markers.

**Conclusion:**

The causal hypotheses tested in this study demonstrate that individuals on LCD with high fibre intakes improved their CVD markers as expected, but those on LCD who increase fat intake had no effects on CVD markers mediated by obesity and diabetes.

**WHAT IS ALREADY KNOWN ON THIS TOPIC**

- High carbohydrate intake can increase cardiovascular disease (CVD) risk factors, while low total carbohydrate intake could have beneficial effects on cardiometabolic risk markers.
- Mediation analysis is a novel approach applied in causal inference and its use to investigate the effect of low carbohydrate diets (LCD) on CVD markers has not been tested.

**WHAT THIS STUDY ADDS**

- The causal hypotheses tested in this study demonstrate that individuals on LCD with high fibre intakes improved their CVD markers as expected.
- However, those on LCD who increase fat intake had no effects on CVD markers mediated by obesity and diabetes.

**HOW THIS STUDY MIGHT AFFECT RESEARCH, PRACTICE OR POLICY**

- The mechanism underlying the significant increase in the high-density lipoprotein cholesterol in people consuming LCD remains unclear.
- Dietary guidance focusing on healthy dietary patterns is more likely to promote cardiovascular health.
- Building a strong evidence base through high-quality observational and intervention studies is critical for effective dietary recommendations.

## Introduction

Cardiovascular disease (CVD) is considered the leading cause of mortality worldwide. In 2019, high systolic blood pressure (SBP) was the leading death risk factor globally, accounted for 10.8 million deaths.^1^ The high fasting plasma glucose and high body mass index (BMI) were two of the major risks exposure.^1^ Unhealthy diets, in particular those characterised by being high in calories, sugar, sodium and trans fats, and low in fibre and micronutrients, are major risk factors for developing CVD.^2 3^ Dietary pattern represents habitually consumed food and nutrients in terms of quantity, variety and combination.^4^ Since food intake is a multidimensional exposure with intercorrelations among different foods, promoting healthier dietary patterns could be a more comprehensive and effective lever than an individual nutrient improvement to overcome the burden of CVD.

In the UK, current guidelines for primary CVD prevention recommend increased intake of whole grains, fruits and vegetables,^5^ all of which are sources of carbohydrates. Carbohydrates can be broadly classified into sugars, starch and fibre, with each having a different impact on health outcomes. While high intake of sugar-sweetened beverages has been associated with increased CVD incidence and mortality,^6^ intake of foods rich in dietary fibre are associated with reduced total cholesterol and low-density lipoprotein (LDL) and improvements in glycaemic control, especially in individuals with overweight and obesity.^7 8^ Low-carbohydrate diets (LCD) (<45 E%) have shown beneficial effects on cardiometabolic risk markers including weight loss and serum lipids.^9^ However, its impact on others clinical endpoints such as myocardial infarction, stroke and overall mortality is still largely unknown.^10^

In the UK, sources of carbohydrates such as ‘cereals and cereal products’ (38%–44% of total fibre intake), followed by ‘vegetables and potatoes’ (21%–32%) and ‘fruit’ (6%–16%) are reported to be the main sources of fibre consumed in all age groups.^11^ Therefore, reduced carbohydrate intake in LCD may be associated with decreased fibre intake or accompanied by increases in fat and/or protein intake, which in turn may affect glucose and lipid metabolism.^9 12^ In this sense, focusing public health messages on the quantity over the quality of carbohydrates could result in unintended and harmful consequences, such as replacement of carbohydrates with high nutritional value (ie, fruits and vegetables) for alternatives of low nutritional value (ie, sugar-sweetened beverages or processed meat).^13^

The use of mediation analysis in nutrition research has become more common. It provides insights into the relationship between the variables in a potential causal chain thus offering a useful tool to indirectly investigate mechanisms in prevention research. In observational studies, causal mediation analysis provides general definitions of causal direct, indirect and total effects, useful for better understanding the mechanisms of exposure-and-outcome effects.^14^ Mediating analyses are, therefore, important in informing health policy decisions, lead to intervention optimisation and guide implementation. However, observational studies do not always use statistical methods to assess mediation which may impact reproducibility, evidence synthesis and implementation.^15 16^

The aim of this study was to examine the associations between different types of LCD and conventional risk factors for CVD and investigate whether these associations are mediated by BMI, waist circumference (WC) and haemoglobin A1c (HbA1c) applying causal mediation analyses.

## Methods

### Design and participants

This study is a secondary analysis of publicly available data from the UK National Diet and Nutrition Survey (NDNS) programme. This is a continuous cross-sectional survey providing high-quality, nationally representative data on food consumption, lifestyle, health information and cardiometabolic risk factors of the general population aged 1.5 years and over living in private households in the UK. A random clustered sample was drawn from the UK Postcode Address File and the survey covers a representative sample of around 1000 people per year. Details on the survey design and sampling methods of the NDNS Rolling Programme (NDNS RP) have been published elsewhere.^17^ Since cardiovascular outcomes more commonly affect older people this study sample includes adults aged 45–80 years old from years 1–4 (2008/2009–2011/12), 5–6 (2012/2013–2013/2014), 7–8 (2014/2015–2016/2016) and 9 (2016/2017) of the UK NDNS programme with data on diet intake, anthropometric measurements and laboratory parameters.

### Data collection procedures

#### Dietary assessment

Dietary assessment was carried out using the means of 4-day estimated food diaries^17^; all participants who completed three or more diary days were included in the survey. Participants were asked to keep a record of everything eaten or drunk over four consecutive days, both at home and away from home. Trained interviewers undertook three visits with each participant. At the first visit, the interviewer placed the diary which was followed by a brief second visit/contact to provide support during completion and check for compliance. At the third visit, the diary was reviewed, edited for possible omissions and collected. Diaries were coded by trained coders and processed using the Diet in Nutrients Out algorithm.^18^ Each recorded item was assigned a suitable food and portion code. The food composition data used were the Department of Health’s NDNS Nutrient Databank and portion sizes from the Food Standard Agency’s portion size book.^19^ Nutrients such as carbohydrate, protein and total fat were expressed as a percentage of total energy intake, while indicators relating to fibre intake were expressed per 1000 kcal. Dietary fibre intake was defined as non-starch polysaccharides as measured by the Englyst method.^20^

#### Anthropometric measurements

Body weight and body height were measured by a scale and by a stadiometer, respectively, during the interview. BMI was calculated by body weight (kg) divided by a square of height (m^2^). Waist and hip circumference was measured by nurses with an insertion tape calibrated in mm.

#### Assessment of HbA1c, lipids and blood pressure

Fasting blood samples were collected for measurements of HbA1c and glucose. The volume of blood collected varied by age, with 33 mL being taken from adults. Blood was collected using an EDTA tube for HbA1c analysis. The EDTA sample was posted by the nurse on the day of collection and HbA1c was usually analysed within 24–48 hours of sampling in the nominated laboratory for the NDNS RP at Addenbrooke’s Hospital in Cambridge, UK. Total and high-density lipoprotein (HDL) cholesterol were measured in blood serum and processed using a Siemens Dimension analyser. LDL was measured indirectly using the Friedewald equation. C reactive protein (CRP) was measured by a high-sensitivity assay. Blood pressure was measured in a sitting position using a validated machine (Omron HEM907). Average SBP was defined as the average of three measurements taken at 1 min intervals. The time between the diet diary recording period and blood sampling was at least 8 weeks in year 2 onwards.

#### Sociodemographic data

Sociodemographic data including age, sex, ethnicity (white, Asian, black, mixed and other) and socioeconomic status (lived in owner-occupied accommodation, social housing or privately rented accommodation) was gathered during face-to-face interviews conducted by the NDNS researchers and used as a control covariates.

#### Data source

The data can be accessed online through the UK Data Service.^21^ ^22^

#### Synthetic treatments

Four synthetic treatment scenarios were explored in this study: (1) LCD, (2) low carbohydrate and high fibre diet (LCHF), (3) low carbohydrate and high saturated fat diet (LCHS) and (4) low carbohydrate and high unsaturated fat diet (LCHU). The LCD was defined as less than 45% of total energy intake from carbohydrates.^23^ LCHFs were considered those with more than 25 g fibre per day^23^; low carbohydrate and high fat diets were those with more than 35% of total energy intake from fat^23^; LCHS meant those diets with more than 35% of total energy intake from fat and more than 10% of total energy intake from saturated fat; and, LCHU was defined as more than 35% of total energy intake from fat and more than 20% of total energy intake from unsaturated fat.^23^ Controls were matched based on propensity scores as explained below.

#### Outcomes of interest

BMI and WC were used as markers of obesity. HbA1c was used as a of glycaemic control; total cholesterol, HDL and LDL cholesterol and triglycerides (TRIG) were markers of dyslipidaemia; SBP and diastolic blood pressure (DBP) were markers of hypertension; CRP was a marker of inflammation. The analyses were adjusted for age (year), sex (male/female), socioeconomic status (based on the employment of the household reference person for their household), smoking (current smokers or not), estimated energy intake (mean kcal), alcohol consumption (units of alcohol per day) and hypertension medication prescribed.

### Statistical analyses

Randomised controlled trials (RCTs) are considered the gold standard approach for estimating the effects of treatments and intervention on outcomes. Given that RCT was not feasible in this context we used observational data and employed regression models with two-stage adjustment to account for differences in measured characteristics in their studies. In this paper, we rely on the potential outcomes framework and average treatment effects (ATEs) introduced by Rubin combined with causal diagrams and domain expert knowledge motivated by Pearl.^24 25^

#### Total, direct and indirect effect

**Figure 1:**
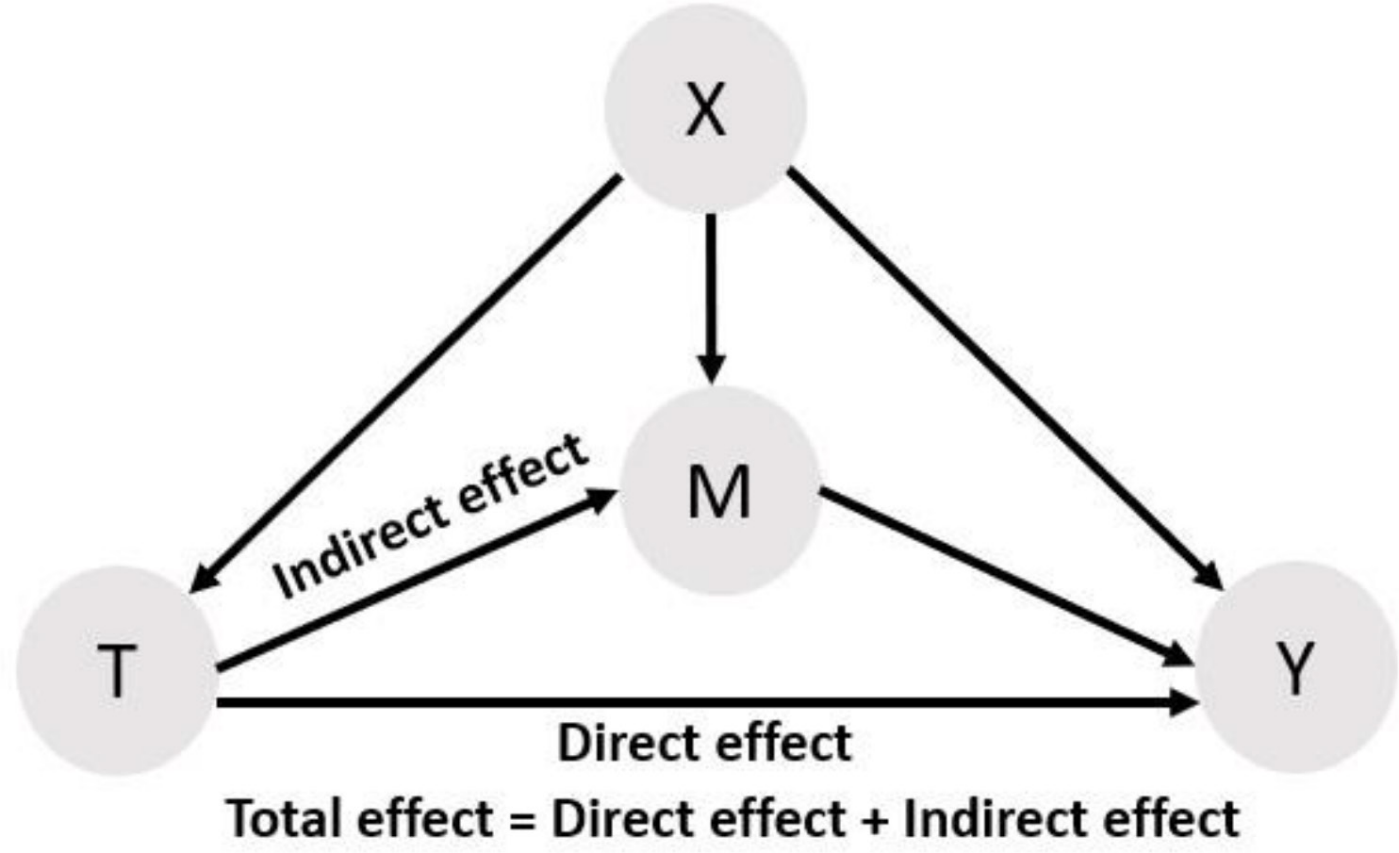
Directed acyclic graph for the causal effect of treatment T on the outcome Y where X is the set of confounders and M is a mediator.

Typically, we aim at identifying the total effect of the treatment on the outcome. This total effect sums the effect of the treatment that acts through a given set of mediators of interest (indirect effect) and the effect of the treatment unexplained by those same mediators (direct effect). It is very common to approach mediation analysis through linear regression by adjusting for the mediator, which allows the estimation of the direct effect.^26^ We use the counterfactual framework to estimate the total and direct effect. The total effect of a treatment T when mediating for M is obtained by not including M a control variable. Alternatively, the direct effect is obtained by including the mediator M in the set of confounders. To illustrate the approach, we used the following directed acyclic graph (figure 1).

The direct effect is represented by the direct arrow T. The indirect effect is the effect that propagates through the pathway T, while the total effect is the summation of the two effects.

To isolate the indirect effect from the direct effect, we need to consider a hypothetical change in the mediator while keeping the treatment constant, which was done by including and excluding the mediator from the set of control variables.

To identify a potential causal effect of LCD on CVD risk (denoted by Y) and produce unbiased estimates that are not affected by other factors (confounders, denoted by X), we estimate the ATE, that is, the difference in average outcomes for the treated and untreated individuals, defined to be $E\left[Y_{i}\left(1\right)-Y_{i}\left(0\right)\right]$ , where $Y_{i}\left(1\right)$ and $Y_{i}\left(0\right)$ are the pair of potential outcomes of individual i . In order to create the counterfactual ( $Y_{i}\left(0\right)$ for a treated individual and $Y_{i}\left(0\right)$ for a controlled individual, we rely on the propensity score,^25^ defined as the probability of treatment assignment conditional on observed baseline characteristics.

The underlying principles of ATEs estimation based on propensity score matching consists of matching treated and untreated individuals (ie, individuals who are following a given diet with individuals who are not and vice versa) in terms of their observable characteristics (X), and then comparing the outcomes (Y) (ie, CVD risk) of those ‘following a particular diet’ and ‘not following a particular diet’ individuals that have the same diet propensity. The matching process also ensures that comparisons between the two groups of individuals occur only between individuals with close observable characteristics X.

Following Becker and Ichino,^27^ we first estimated the propensity score, that is, the probability of receiving a treatment, given the observed pretreatment characteristic we want to control of the effect of (such as age, sex, ethnicity, socioeconomic status, smoking status, alcohol intakes and total calories intakes), using a logistic regression model $e_{i}\left(X_{i}\right)=P\left(T_{i}=1|X_{i}\right)$ . We then matched each individual from the treated group (respectively, control group) to its closest counterpart (twin) from the opposite group using k-nearest neighbours. The difference in the outcome between the ‘on-diet’ and an average of the outcome of its matches was then calculated for each individual. Finally, ATE was obtained by averaging these differences across all the matches M:

$$ATE=\frac{1}{M}\sum_{i=1}^{M}\left(Y_{i}-\frac{1}{|J_{i}|}\sum_{j\in J_{i}}Y_{j}\right)$$

where $J_{i}$ is the set of untreated (resp. treated) individuals matched to the treatment (respectively, control) individual i , and |.| is the number of elements in the set.

The quantity we are estimating is a weighted average of the average treatment of the treated and average treatment of the control each having the same formula as ATE but where the matches focus on one of the groups.

In addition to the estimate of the ATE, we estimated the significance (p values) and CI. The p value was calculated as the empirical probability of obtaining results at least as extreme as the test statistic given that the null hypothesis is true. The null hypothesis is that ‘CVD risk is the same across the two groups’. To test this null hypothesis, we perform a permutation which results in the equivalent question of whether ‘the labels assigning samples to outcomes are interchangeable’, in other words, ‘Is the distribution of outcomes the same for all groups even if we randomly reassign the treatment?’. A p<0.05 indicates that the labels are not interchangeable and that the original label configuration is relevant with respect to the data. We also calculated 95% CIs using the bootstrapping method using 1000 subsamples.^28^ We used for our estimation of the ATE the Python package DoWhy^29^ and built our permutation analysis on top of the package to compute the p-values of confidence intervals.

## Results

Table 1 shows participant characteristics. The original data had 13 350 observations for body measurements and 7950 for dietary information. Between 2008 and 2016, a total of 3640 participants met inclusion criteria (individuals with age between 40 and 85 years and drop observations with missing data) and were included in the analysis. Of these, most were women (56.8%) and the mean (±SD) age was 59.97 (±9.89) years. N=1336 (36.7%) participants were overweight and N=1111 (30.5%) were obese. Twelve participants (0.3%) had a known diagnosis of diabetes.

**Table 1:** Characteristics of participants

	N (%)	Mean (SD)	Cut-off points
Age		59.97 (9.89)	
Sex			
Male	1572 (43.19)		
Female	2068 (56.81)		
Current smoker	665 (18.27)		
Use of hypertension medication	2786 (76.5)		
Body mass index			
Normal weight	875 (24)		<25 kg/m^2^
Overweight	1336 (36.7)		≥25, <30 kg/m^2^
Obesity I	750 (20.6)		≥30, <35 kg/m^2^
Obesity II	262 (7.2)		≥35, <40 kg/m^2^
Obesity III	99 (2.7)		≥40 kg/m^2^
Waist circumference	2720 (74.7)	96.72 (14.47)	>94 cm(M); >80 cm (W)
Waist-to-hip ratio		0.902 (0.09)	≥0.90 (M); ≥0.85 (W)
Normal weight	1193 (32.8)		
Overweight	978 (26.9)		
Obesity	545 (15.0)		
Diabetes status			
No diabetes	2064 (56.7)		
Pre-diabetes	170 (4.7)		
Diabetes	12 (0.3)		
Triglycerides		53.71 (34.06)	
Desirable	1940 (53.3)		<150 mmol/L
Borderline	12 (0.3)		150–200 mmol/L
High	2 (0.1)		>200 mmol/L
Cholesterol		203.02 (44.12)	
Desirable	902 (24.8)		<200 mmol/dL
Borderline	696 (19.1)		200–239 mmol/dL
High	363 (10.0)		>240 mmol/dL
HDL		56.11 (17.62)	<40 mmol/dL
LDL		123.96 (39.32)	>100 mmol/dL
Systolic blood pressure		130.95 (17.62)	
Desirable	1993 (54.8)		<140 mmHg
High	762 (20.9)		≥140 mm Hg
Diastolic BP		75.54 (11.2)	
Desirable	2490 (68.4)		<90 mm Hg
High	265 (7.3)		≥90 mm Hg
CRP (mg/L)		3.94 (6.04)	<5 mg/L
Energy intake (kcal)		1629.94 (496.53)	
% energy from carbohydrate (kcal/food kcal)		47.2 (6.8)	
% energy from total sugars (kcal/food kcal)		20.2 (6.8)	
% energy from protein (kcal/food kcal)		17.7 (3.7)	
% energy from fat (kcal/food kcal)		35 (6.6)	
% energy from saturated fat acids (kcal/food kcal)		13.3 (3.6)	
Fibre intake g/day		18.5 (6.76)	

BP, blood pressure; CRP, C reactive protein; HDL, high-density lipoprotein; LDL, low-density lipoprotein.

The mean (±SD) total energy intake was 1629.94 (±496.5 kcal). Mean (±SD) carbohydrate, protein, total fat and saturated fat intake was approximately 47% (±6.8), 18% (±3.7), 35% (±6.6) and 13% (±3.6) of food energy across participants. The average intake of fibre was 18.5 g/day. Table 2 shows the percentage of carbohydrate, fibre, saturated fat and unsaturated fat intake in total energy intake for each synthetic treatment group.

**Table 2:** Percentage energy intake from carbohydrates, saturated fats and unsaturated fats and total fibre intake for each of the control and synthetic treatment groups

	Low CHO		Low CHO HFibre		Low CHO HSat		Low CHO HUnSat	
	Control mean (SD)	Treatment mean (SD)	Control mean (SD)	Treatment mean (SD)	Control mean (SD)	Treatment mean (SD)	Control mean (SD)	Treatment mean (SD)
Carbohydrates (%)	51 (4.6)	40.2 (4)	47.5 (6.7)	40.9 (4.5)	50.5 (5)	40.0 (4.1)	49.4 (5.6)	39.2 (4.5)
Saturated fat (%)	12 (3)	15.4 (3.4)	13.2 (3.6)	14.5 (3.2)	12 (3)	16 (3)	12.7 (3.0)	15.2 (3.2)
Unsaturated fat (%)	16.8 (3.2)	21.3 (3.8)	18.2 (3.9)	22.9 (4.9)	16.9 (3.4)	21.7 (3.5)	17 (3.0)	23.6 (3.1)
Fibre (g/day)	19.11 (7)	17.36 (6.2)	18 (6.5)	29 (4.2)	19 (7)	17.3 (6.1)	18.7 (6.8)	17.8 (6.5)

As shown in table 3, the mediated (indirect) effects of BMI, BMI and WC or BMI, WC and HbA1c were not significant for the associations between LCD and CVD markers, with the exception of a positive effect on TRIG observed in model 3 (p<0.01). This effect was fully mediated by BMI, WC and HbA1c as this relationship was not significant when we looked at the direct effects, indicating that HbA1c plays an important role in TRIG increase. LCD had a small positive direct effect on HbA1c in model 1 only (p<0.05), demonstrating an increase in HbA1c even in individuals who maintained their BMI. It also had a positive effect on HDL in models 2 (p<0.01) and 3 (p<0.01), and on SBP in model 3 (p<0.05).

**Table 3:** Direct and indirect effects of low carbohydrate diet on markers of CVD risks with BMI, WC and HbA1c as mediators among UK adults aged 45–80 years in NDNS (2008/2009–2016/2017)

Mediator and outcomes	Direct effect		Indirect effect	
	Estimate	95% CI	Estimate	95% CI
BMI as mediator (model 1)				
HbA1c	0.07	−0.03 to 1.13**	0.02	−0.1 to 0.12
TRIG	−0.67	−3.45 to 2.9	1.04	−4.65 to 4.63
TC	0.67	−2.15 to 5.56	1.07	−5.95 to 6.09
HDL	0.97	0.39 to 3.57	−0.66	−2.73 to 2.67
LDL	−1.53	−4.34 to 3.74	1.48	−6.02 to 5.48
SBP	0.46	−0.38 to 2.76	−0.9	−2.37 to 2.4
DBP	0.66	−0.1 to 1.71	−0.03	−1.51 to 1.37
CRP	−0.25	−0.85 to 0.2	0.08	−0.79 to 0.78
BMI and WC as mediators (model 2)				
HbA1c	0.06	−0.02 to 0.13	−0.02	−0.1 to 0.11
TRIG	−1.95	−3.3 to 2.99	2.27	−4.55 to 4.75
Total Chol	3.07	−1.49 to 6.8	0.03	−5.97 to 6.53
HDL	2.12	0.43 to 3.58***	−0.29	−2.46 to 2.97
LDL	−0.04	−3.89 to 3.78	0.85	−5.64 to 5.26
SBP	1.1	−0.27 to 2.63	−1.06	−1.99 to 2.69
DBP	0.74	−0.23 to 1.58	−0.2	−1.46 to 1.54
CRP	−0.19	−0.72 to 0.29	0.0	−0.69 to 0.83
BMI, WC, HbA1c as mediators (model 3)				
TRIG	1.75	−3.49 to 3.12	3.68	−4.72 to 4.75***
Total chol	1.24	−2.17 to 6.95	−0.51	−5.92 to 6.5
HDL	2.63	0.32 to 3.54***	−0.01	−2.31 to 2.52
LDL	−0.74	−3.59 to 3.85	−0.37	−5.5 to 5.03
SBP	1.67	0.83 to 4.07**	−1.12	−2.17 to 2.46
DBP	0.91	0.14 to 2.11	−0.06	−1.54 to 1.44
CRP	−0.31	−0.57 to 0.45	−0.22	−0.67 to 0.83

The confounders for the analysis are age, sex, ethnicity, economic status, smoking status, total calories intake, alcohol intake and whether taking general medicines.

Indirect effect: the effect of diet on the outcome that is explained by the mediator.

Direct effect: the effect of diet on the outcome that is not explained by the mediator.

**p≤0.05, ***p<0.01.

BMI, body mass index; CRP, C reactive protein; CVD, cardiovascular disease; DBP, diastolic blood pressure; HbA1c, glycated haemoglobin; HDL, high-density lipoprotein; LDL, low-density lipoprotein ; NDNS, National Diet and Nutrition Survey; SBP, systolic blood pressure; TC, total cholesterol; TRIG, triglycerides; WC, waist circumference.

In table 4, positively mediated effects for the association between LCHF and CRP were consistently observed across the three models (p<0.01). LCHF also had positive indirect effect on TRIG in models 2 (p<0.05) and 3 (p<0.01) but a negative indirect effect on LDL in models 1 and 2 (p<0.01) and 3 (p<0.05). LCHF had a negative direct effect on SBP in the three models (p<0.01) and DBP in models 2 (p<0.01) and 3 (p<0.05), decrease TRIG in model 1 (p<0.01) and decrease TC model 3 (p<0.05).

**Table 4:** Direct and indirect effects of low carbohydrate and high fibre diet consumption on markers of CVD risks with BMI, WC and HbA1C as mediators among UK adults aged 45–80 years in NDNS (2008/2009–2016/2017)

Mediator and outcomes	Direct effect		Indirect effect	
	Estimate	95% CI	Estimate	95% CI
BMI as mediator (model 1)				
HbA1c	0.48	−0.01 to 0.59***	0.33	−0.49 to 0.55***
TRIG	−8.43	−14.61 to −0.61***	4.92	−7.62 to 11.04
Total Chol	−21.46	−25.57 to 12.37***	−9.68	−35.76 to 25.32***
HDL	−2.82	−7.28 to 9.6	−1.2	−16.51 to 10.93
LDL	−8.17	−18.88 to 8.28	−12.0	−26.13 to 17.88***
SBP	−5.08	−9.05 to 0.54***	0.55	−5.57 to 9.36
DBP	−0.64	−6.95 to 1.04	0.22	−6.34 to 6.08
CRP	0.81	−1.21 to 1.86	1.46	−2.51 to 2.48***
BMI and WC as mediators (model 2)				
HbA1c	0.38	−0.08 to 0.62***	0.24	−0.54 to 0.58***
TRIG	−3.44	−13.37 to −0.09	6.32	−6.67 to 13.05**
Total chol	−22.24	−25.05 to 14.85***	−10.24	−35.46 to 32.21**
HDL	−6.78	−7.75 to 8.82***	−7.22	−17.31 to 9.4***
LDL	−9.77	−18.16 to 11.18**	−11.48	−24.64 to 25.73***
SBP	−4.91	−9.4 to 1.14***	0.58	−5.9 to 9.29
DBP	−4.4	−6.18 to −0.54***	−0.67	−5.57 to 6.37
CRP	1.28	−0.94 to 1.83	1.23	−2.38 to 2.27**
BMI, WC, HbA1c as mediators (model 3)				
HbA1c	NA	NA	NA	NA
TRIG	−3.91	−14.46 to 0.49	8.79	−6.27 to 13.18***
Total Chol	−11.61	−29.13 to 14.58**	−7.31	−34.77 to 24.64
HDL	−3.66	−8.3 to 7.37**	−6.09	−16.57 to 9.07***
LDL	−7.24	−18.01 to 16.64	−7.86	−26.45 to 22.31**
SBP	−6.46	−9.23 to 1.4***	0.53	−4.89 to 10.55
DBP	−2.88	−7.06 to 0.97**	0.45	−5.83 to 6.27
CRP	1.26	−1.07 to 1.61	1.55	−2.52 to 2.33***

The confounders for the analysis are age, sex, ethnicity, economic status, smoking status, total calories intake, alcohol intake and whether taking general medicines.

Indirect effect: the effect of diet on the outcome that is explained by the mediator.

Direct effect: the effect of diet on the outcome that is not explained by the mediator.

**p≤0.05, ***p<0.01.

BMI, body mass index; CRP, C reactive protein; CVD, cardiovascular disease; DBP, diastolic blood pressure; HbA1c, glycated haemoglobin; HDL, high-density lipoprotein ; LDL, low-density lipoprotein; NA, not available; NDNS, National Diet and Nutrition Survey; SBP, systolic blood pressure; TC, total cholesterol; TRIG, triglycerides; WC, waist circumference.

In table 5, indirect effects of LCHS were only observed in model 1 where LCHS was associated with elevated HbA1c and low TRIG, LDL and CRP. Positive direct effects were observed for HDL in models 1 (p<0.05) and 3 (p<0.01) and for HbA1c in model 2 (p<0.05). Positive direct effects were observed for SBP across the three models, more significant in model 3 (p<0.01).

**Table 5:** Direct and indirect effects of low carbohydrate diet and high saturated fat consumption on markers of CVD risks with BMI, WC and A1C as mediators among UK adults aged 45–80 years in NDNS (2008/2009–2016/2017)

Mediator and outcomes	Direct effect		Indirect effect	
	Estimate	95% CI	Estimate	95% CI
BMI as mediator (model 1)				
HbA1c	0.05	−0.0 to 0.16	0.05	−0.11 to 0.11**
TRIG	−2.47	−4.8 to 1 72	−3.58	−4.56 to 4.79***
Total chol	2.36	−1.93 to 7.03	3.17	−6.55 to 6.45
HDL	1.94	0.5 to 3.92**	1.06	−2.46 to 2.29
LDL	1.31	−3.04 to 4.41	−3.16	−5.87 to 5.43**
SBP	1.36	−0.03 to 3.08**	−1.66	−2.49 to 2.42**
DBP	0.67	−0.52 to 1.31	−0.19	−1.59 to 1.41
CRP	−0.2	−0.63 to 0.41	−0.41	−0.75 to 0.75**
BMI and WC as mediators (model 2)				
HbA1c	0.1	0.0 to 0.16**	0.02	−0.1 to 0.12
TRIG	−0.78	−4.09 to 2.44	−2.28	−4.81 to 4.77
Total chol	2.49	−1.27 to 7.28	−0.01	−6.12 to 6.46
HDL	1.35	0.54 to 3.86	−0.36	−2.59 to 2.33
LDL	2.1	−3.07 to 4.72	−1.44	−5.57 to 5.68
SBP	1.36	−0.08 to 2.93**	−0.11	−1.78 to 3.05
DBP	0.1	−0.49 to 1.23	0.33	−1.52 to 1.44
CRP	0.04	−0.56 to 0.51	−0.05	−0.68 to 0.9
BMI, WC, HbA1c as mediators (model 3)				
HbA1c	NA	NA	NA	NA
TRIG	−1.54	−4.36 to 2 47	−2.51	−4.78 to 5.11
Total chol	1.86	−0.89 to 8.18	−0.66	−6.28 to 5.78
HDL	2	0.55 to 4.24***	0.83	−2.51 to 2.57
LDL	−0.74	−3.03 to 4.66	−2.65	−6.23 to 5.65
SBP	2.99	1.02 to 4.47***	−0.11	−1.84 to 2.91
DBP	0.79	−0.43 to 1.67	0.12	−1.34 to 1.54
CRP	0.01	−0.44 to 0.56	−0.21	−0.65 to 0.85

The confounders for the analysis are age, sex, ethnicity, economic status, smoking status, total calories intake, alcohol intake and whether taking general medicines.

Indirect effect: the effect of diet on the outcome that is explained by the mediator.

Direct effect: the effect of diet on the outcome that is not explained by the mediator.

**p≤0.05, ***p<0.01.

BMI, body mass index; CRP, C reactive protein; CVD, cardiovascular disease; DBP, diastolic blood pressure; HbA1c, glycated haemoglobin; HDL, high-density lipoprotein; LDL, low-density lipoprotein; NA, not available; NDNS, National Diet and Nutrition Survey; SBP, systolic blood pressure; TC, total cholesterol; TRIG, triglycerides; WC, waist circumference.

In table 6, mediated effects for the association between LCHU and CVD markers were only observed in model 3, where BMI, WC and HbA1c only mediated the effect of the LCHU on total cholesterol and DBP. In addition, LCHU had a consistent positive direct effect on HDL across models. Positive direct effects in CRP were only observed in model 1 (p<0.05).

**Table 6:** Direct and indirect effects of low carbohydrate diet and high unsaturated fat consumption on markers of CVD risks with BMI, WC and A1C as mediators among UK adults aged 45–80 years in NDNS (2008/2009–2016/2017)

Mediator and outcomes	Direct effect		Indirect effect	
	Estimate	95% CI	Estimate	95% CI
BMI as mediator (model 1)				
HbA1c	0.04	−0.04 to 0.14	−0.05	−0.12 to 0.11
TRIG	−1.89	−5.18 to 1.8	−0.23	−5.47 to 5.66
Total chol	0.3	−1.76 to 7.82	1.44	−6.62 to 6.27
HDL	3.16	0.82 to 4.65***	−1.17	−2.46 to 2.49
LDL	0.55	−3.61 to 4.56	0.08	−5.82 to 6.53
SBP	1.09	−0.73 to 2.5	−0.06	−2.57 to 2.91
DBP	0.8	−0.36 to 1.62	0.4	−1.54 to 1.9
CRP	−0.59	−0.85 to 0.25**	0.3	−0.77 to 0.86
BMI and WC as mediators (model 2)				
HbA1c	0.02	−0.05 to 0.14	−0.06	−0.12 to 0.13
TRIG	−1.89	−4.89 to 2.1	−2.26	−4.8 to 5.55
Total chol	2.41	−1.36 to 9.03	3.23	−6.64 to 6.71
HDL	2.19	0.83 to 4.47**	−1.09	−2.77 to 2.81
LDL	0.54	−3.13 to 5.36	0.22	−6.05 to 6.72
SBP	0.81	−0.7 to 2.47	0.71	−2.54 to 2.98
DBP	0.7	−0.33 to 1.73	0.5	−1.69 to 1.83
CRP	−0.17	−0.75 to 0.35	−0.4	−0.77 to 0.86
BMI, WC, HbA1c as mediators (model 3)				
HbA1c	NA	NA	NA	NA
TRIG	−0.48	−5.36 to 2.06	−0.65	−5.03 to 5.99
Total chol	6.72	−1.03 to 9.0**	6.52	−7.4 to 6.73***
HDL	3.26	0.87 to 4.73***	−0.51	−2.82 to 2.72
LDL	1.26	−3.37 to 5.97	0.57	−6.05 to 6.21
SBP	1.85	0.27 to 4.05	0.37	−2.48 to 2.92
DBP	1.26	−0.45 to 2.05**	1.13	−1.57 to 1.98**
CRP	0.0	−0.74 to 0.47	0.29	−0.74 to 0.87

The confounders for the analysis are age, sex, ethnicity, economic status, smoking status, total calories intake, alcohol intake and whether taking general medicines.

Indirect effect: the effect of diet on the outcome that is explained by the mediator.

Direct effect: the effect of diet on the outcome that is not explained by the mediator.

**p≤0.05, ***p<0.01.

BMI, body mass index; CRP, C reactive protein; CVD, cardiovascular disease; DBP, diastolic blood pressure; HbA1c, glycated haemoglobin; HDL, high-density lipoprotein; LDL, low-density lipoprotein ; NDNS, National Diet and Nutrition Survey; SBP, systolic blood pressure; TC, total cholesterol; TRIG, triglycerides; WC, waist circumference.

## Discussion

In this study, we used a nationally representative sample of UK adults to explore the effects of LCDs on CVD risk markers, direct and mediated by obesity or glycaemia. We observed that the triad of BMI, WC and HbA1c fully mediated the association between LCD and triglycerides. Although they also fully mediated the effects of LCHF on LDL, BMI and WC were sufficient to fully mediate the effects of LCHF on triglycerides and CRP. In addition, BMI alone fully mediated the effects of LCHS on HbA1c, triglycerides, LDL and CRP. None of these mediators seem to explain the effect of LCHU on CVD risk markers, such as HDL and CRP.

Our findings are in keeping with previous studies which demonstrated that certain characteristics, such as BMI and glucose metabolism, can influence individuals’ susceptibility to triglyceride changes following dietary carbohydrate intake.^29^ Although the direction of the association between low carbohydrate intake and triglycerides in our study was not in agreement with previous studies where LCD resulted in reduced triglyceride levels,^9 10^ the width of our confidence suggests that we cannot be certain about the effect observed. Previous studies have shown that simple sugars can have more deleterious effects on triglyceride levels compared with starches, which was not assessed in this study.^30^

Also, LCHF was associated with lower LDL, SBP and DBP, which agree with previous studies on the impact on dietary fibre on CVD risk.^31^ The effect on LDL was fully mediated by general adiposity and glucose levels. Obesity is a well known risk factor for high LDL levels.^32^ On the other hand, the link between glucose control and LDL is thought to be more complex as dyslipidaemia can be the cause or consequence of disturbances of glucose metabolism.^33^

A previous meta-analysis has shown that individuals in the lowest quintile of carbohydrate intake in addition to consuming lower than average dietary fibre, also consumed more animal fat than individuals in the other quintiles.^34^ Animal fats are a common source of saturated fats in the diet. Substitution of carbohydrate by saturated fatty acids has been associated with increased total cholesterol, LDL and HDL concentrations,^9^ yet there is little to no evidence on the impact of saturated fat intake on HDL and triglycerides, and only small reductions total cholesterol and LDL cholesterol and BMI.^35^ In our study, LCHS was associated with higher HDL and lower LDL, yet confidence intervals were wide for all estimates. BMI seemed to play an important mediation role on the effect of LCHS on CVD risk. However, the effects of LCHU on CVD risk factors did not seem to be mediated by BMI, WC and HbA1c.

Research landscape has evolved from examining individual foods and nutrients to using dietary patterns to represent the combined effects of foods and beverages on different diets. Dietary pattern analysis considers complex interactions between nutrients and foods, as well as the cumulative effect of the overall diet, which may be more powerful than the individual effect of each nutrient.^36^ Existing evidence suggests that to achieve better cardiovascular health, individuals should choose an overall healthy eating pattern that emphasises the quality and healthy sources of carbohydrates and fats, rather than the absolute amounts of fats or carbohydrates in the diet.^37^

Dietary pattern rather than isolated nutrients has increasingly explained the relationship between diet and CVD. Widely consolidated in the literature, a dietary pattern based on fruits, vegetables, whole grains, nuts, fish and liquid vegetable oils has been associated with better overall health outcomes and decreased risk of CVD.^3–38^

Several limitations need to be considered when interpreting our findings. It is known that people diagnosed with CVD are often advised to change their diet and lifestyle to a healthier pattern,^39^ which can be evidenced in the relatively low carbohydrate consumption and satisfactory fibre consumption in the general population in this study. This, in turn, makes the observed associations investigated difficult to interpret. As with any cross-sectional data residual confounding and reverse causation is possible. The possibility of confounding was addressed through statistical adjustment for a wide range of covariates, however, genetic factors, lifestyle (physical activity) and environmental factors were not considered due to the low number of observations on these variables. The low number of observations in some cells also resulted in wide CIs, which makes interpretation and extrapolation of these findings difficult. Ultimately, subgroup analysis based on different types of sugars (naturally occurring vs added), fibre (soluble or insoluble), polyunsaturated fats (omega-3 and omega-6) and protein (animal or vegetable) were not investigated. These nuances could modify associations between macronutrient intake and outcomes. The dietary assessments in this study are from food diaries, therefore, they may not accurately depict typical participants’ intake and, although less likely, may be subject to recall bias.

## Conclusion

The potentially causal relationships assessed in this study demonstrate that individuals on LCD with high fibre intakes improved their CVD markers as expected, but adhering to LCDs with high fat intake had no obesity-mediated or diabetes-mediated effects on CVD markers. The mechanism underlying the significant increase in the HDL-C in people consuming LCD remains unclear and more research on the underlying mechanism is needed. Building a strong evidence base through high-quality observational and intervention studies is critical for effective dietary recommendations.

## Data Availability

Data are available in a public, open access repository.
